# Application of Synchrotron Radiation in Fundamental Research and Clinical Medicine

**DOI:** 10.3390/biomedicines13061419

**Published:** 2025-06-10

**Authors:** Chao Xiao, Jinde Zhang, Yang Li, Mingyuan Xie, Dongbai Sun

**Affiliations:** 1 School of Physics & Southern Marine Science and Engineering Guangdong Laboratory (Zhuhai), Sun Yat-sen University, Guangzhou 510275, China; xiaoch33@mail.sysu.edu.cn; 2 School of Life Sciences, Sun Yat-sen University, Guangzhou 510275, China; 3 School of Medicine, The Chinese University of Hong Kong, Shenzhen 518172, China; zhangjinde@cuhk.edu.cn; 4 Instrumental Analysis and Research Center, Sun Yat-sen University, Guangzhou 510275, China; liyang223@mail.sysu.edu.cn; 5 School of Materials Science and Engineering & Southern Marine Science and Engineering Guangdong Laboratory (Zhuhai), Sun Yat-sen University, Guangzhou 510006, China

**Keywords:** synchrotron radiation X-ray, medical imaging, radiotherapy

## Abstract

Synchrotron radiation light sources have been successfully utilized in material science, biomedicine, and other fields due to their high intensity, excellent monochromaticity, coherence, and collimation. In recent years, synchrotron radiation has significantly expedited the advancement of medical applications, particularly through innovations in imaging and radiotherapy. For instance, synchrotron X-ray imaging has enabled high-contrast and spatial–temporal resolution images for early-stage diagnosis of breast cancer and cardiovascular diseases, offering superior diagnostic accuracy compared to conventional methods. Additionally, novel synchrotron radiation-based radiotherapy techniques, such as microbeam therapy and stereotactic radiotherapy, have shown great potential for clinical application by enabling precise tumor targeting while minimizing damage to surrounding healthy tissues. These advancements are projected to redefine imaging diagnostics and therapeutic strategies, particularly for resistant cancers, by offering enhanced precision, reduced radiation doses, and improved therapeutic outcomes. This review provides an overview of synchrotron radiation beamline characteristics, recent breakthroughs in imaging and radiotherapy, and their emerging applications in treating heart, breast, lung, bone, and brain conditions.

## 1. Introduction

**Figure 1:**
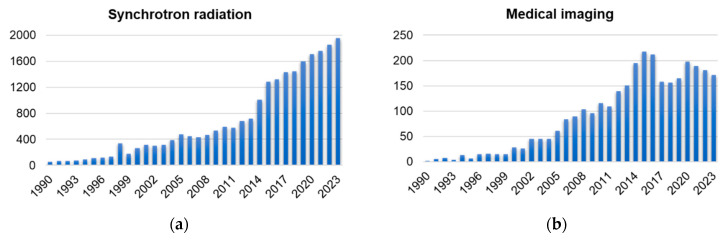

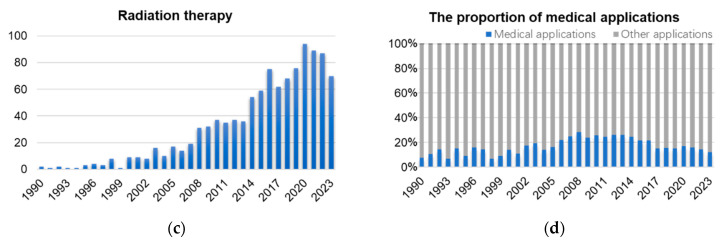
Outcome of synchrotron radiation research in medical imaging and radiotherapy (January 1990 to December 2023). (**a**) Synchrotron radiation. (**b**) Medical imaging. (**c**) Radiation therapy. (**d**) The proportion of medical applications in synchrotron radiation. Data source: PubMed.

Synchrotron radiation, first observed in 1947, refers to the electromagnetic radiation emitted by charged particles traveling near the speed of light along a curved trajectory in a magnetic field [1]. Since its discovery, synchrotron radiation has transitioned from a fundamental physical phenomenon to an indispensable research tool. The X-ray beam generated by synchrotron radiation exhibits exceptional stability, high flux, and a high degree of coherence, making it a unique and versatile tool in advanced research and technology. These properties have facilitated breakthroughs in diverse fields, including biomedical imaging, semiconductor fabrication, and advanced aerospace materials. Over the decades, synchrotron radiation facilities worldwide have undergone continuous evolution, progressing from first-generation to fourth-generation sources. This expansion has led to the establishment of specialized beamlines and cutting-edge technologies designed to tackle increasingly complex scientific and industrial challenges. The Super Photon Ring-8 GeV (SPring-8), the European Synchrotron Radiation Facility (ESRF), and the Advanced Photon Source (APS) are examples of third-generation light sources that have been planned and developed sequentially since the 1990s. These sources are still operating steadily and maintaining high-quality output, which demonstrates the long-term viability of those large-scale research facilities [2]. There is a broad trend toward updating current facilities and constructing new fourth-generation synchrotron sources, such as MAX IV in Sweden, Sirius in Brazil, and the European Synchrotron Radiation Facility Extremely Brilliant Source (ESRF-EBS) in France [3]. Fourth-generation synchrotron sources utilize diffraction-limited storage rings, which minimize electron beam emittance and produce X-ray beams with exceptional brightness and coherence [4,5]. In China, the Beijing Synchrotron Radiation Facility (BSRF), Hefei Light Source (HLS), Shanghai Synchrotron Radiation Facility (SSRF), Taiwan Synchrotron Radiation Facility (TLS), and Taiwan Photon Source (TPS) are all ranked in the middle–low-energy region, and the high-energy light source (High Energy Photon Source, HEPS) with energy exceeding 40 keV is currently under construction. Biomedical applications for medical imaging and radiotherapy are an important part of synchrotron radiation light sources. In terms of imaging, based on the coherence and monochromaticity of synchrotron radiation beams, the capacity of X-ray phase-contrast imaging (PCI) to extract electron density pictures enhances contrast and resolution of imaging results significantly. In radiotherapy, the synchrotron radiation X-ray has a higher flux density than that of a conventional broadband beam. The beam with adjustable energy can accommodate the variations in X-ray absorption by various tissues. Synchrotron radiotherapy can ablate tumor tissues selectively in a more accurate, efficient, and responsible way. The consequence of fundamental scientific research and industrial application from synchrotron radiation has been developing progressively in recent years, as statistical results of PubMed Database reports (1990–2021) show (Figure 1). Compared with the 1990s, the scientific output by the year 2021 has expanded by more than 30 times (Figure 1a). Among these, the research achievements in medical imaging and radiotherapy made possible by synchrotron radiation have significantly influenced the growth of the discipline. As shown in Figure 1b, research activities related to medical imaging exhibited a substantial growth trend initially but have experienced a decline in recent years. In contrast, research related to radiotherapy (Figure 1c) continues to exhibit steady growth. In addition, studies of medical applications have accounted for 15–25% of synchrotron radiation applications since 2000. In the field of biological sciences, synchrotron radiation is gaining importance. In this review, we summarize the development of experimental stations for medical imaging and radiotherapy in synchrotron radiation facilities. Furthermore, we discuss the existing challenges in medical applications and speculate on their future potential.

## 2. Light Sources and Biomedical Beamlines

Several synchrotron-based biomedical beamlines have been constructed throughout the globe, which provide a high-quality research platform for improving the accuracy of disease diagnosis and promoting radiotherapy programs. At present, there are several well-established beamlines in synchrotron radiation facilities, including ESRF (ID17) in France, the Imaging and Medical Beamline (IMBL) in Australia, the BioMedical Imaging and Therapy (BMIT) in Canada, the Synchrotron Radiation for Medical Physics (SYRMEP) of Elettra Sincrotrone Trieste in Italy, BL20B2 of the Super Photon ring-8 GeV (SPring-8) in Japan, 2-BM-A, B of the Advanced Photon Source (APS) in America, etc. Medical-related applications in beamlines are a significant component of synchrotron radiation facilities. It results in enhanced imaging performance due to the synchrotron radiation’s X-ray monochromaticity and energy tunability. Table 1 summarizes the technical characteristics of global active beamlines and their typical medical application cases. Different medical beamlines establish their advantageous imaging and therapeutic applications for diverse research items based on performance variances in beam energy, spot size, flux, and other parameters. For instance, beamlines optimized for higher energy ranges excel in deep tissue imaging, while those with smaller spot sizes are better suited for high-resolution imaging of microscopic structures. These differences highlight the trade-offs in suitability for specific biomedical applications. Furthermore, the Australian Synchrotron (IMBL) excels in high-energy applications (up to 250 keV), making it suitable for lung function imaging and mammography, while the Canadian Light Source (BMIT) offers beamlines optimized for cytochemical visualization and strontium distribution detection with flux densities up to 10^12^ ph/s/mm^2^. Such performance differences define the suitability of each facility for specific medical imaging and therapeutic research.

There are multiple areas of study at different stages in the field of medical applications utilizing synchrotron X-ray imaging (Table 1). Breast cancer imaging, vascular imaging, and skeletal microstructure imaging are all in clinical trials. Preclinical trials have yielded encouraging results, and these procedures are currently being tested on human individuals. Synchrotron X-ray radiation treatment, on the other hand, is still in the animal model stage. This technique has shown promise for precise targeting cancer treatment, but further study is needed to assess its safety and efficacy in humans. Overall, synchrotron X-ray imaging techniques can potentially revolutionize medical diagnosis and therapy; however, further comprehensive research and clinical trials are needed to evaluate their potential benefits and safety completely.

## 3. Synchrotron X-Ray in Medical Imaging

**Figure 2:**
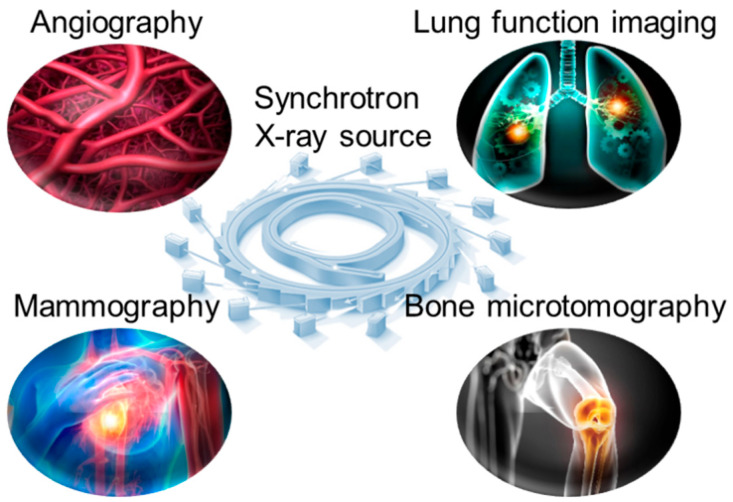
Common medical imaging applications of synchrotron X-ray source.

Traditional X-ray computed tomography (CT) has been widely used in the clinic for disease diagnostic imaging. However, the spectral breadth and intensity of an X-ray beam produced by a conventional source continue to restrict image quality. Photon absorption differences in human tissues reduce contrast in conventional CT imaging. Synchrotron X-ray imaging addresses this with high monochromaticity and energy tunability, optimizing tissue contrast and minimizing beam hardening artifacts. Correcting artifacts using complex mathematical models is one of the most challenging tasks in CT image reconstruction. It is, nevertheless, dependent on complicated algorithms and computational resources. When using synchrotron radiation-based CT imaging, these risks may not occur. When monochromatic synchrotron X-rays are used to irradiate tissue, only the intensity varies. As a result, it successfully avoids the problem of artifacts in medical imaging induced by X-ray beam hardening. Furthermore, the application of sophisticated data-gathering methods and reconstruction algorithms can greatly reduce the number of projections required for CT imaging, hence lowering radiation dosage. In recent years, medical imaging applications employing synchrotron radiation X-ray have expanded and made significant scientific progress. We describe recent practical applications of synchrotron X-ray in angiography, including angiography, lung function imaging, mammography, and bone microtomography (Figure 2).

### 3.1. Angiography

**Figure 3:**
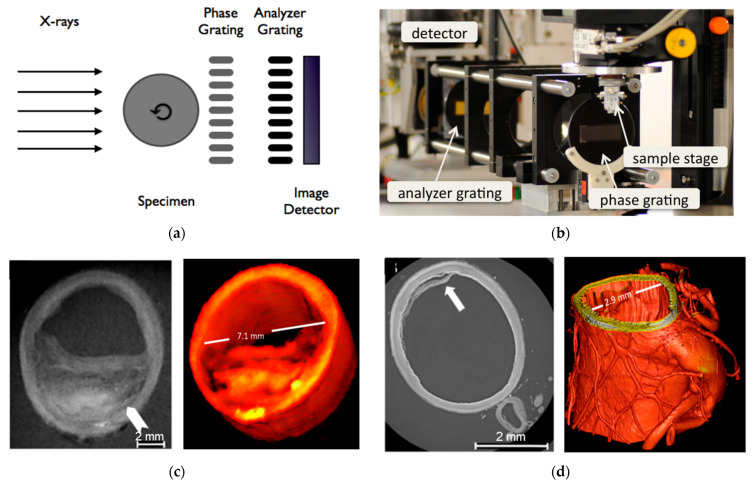
(**a**,**b**) Schematic drawing and photograph of the set-up at the synchrotron beamline ID19, ESRF, France. (**c**) Conventional X-ray cross-sectional image of carotid artery with atherosclerotic plaques (arrow) and its 3D reconstruction image. (**d**) Synchrotron X-ray cross-sectional image of distal external carotid artery (arrow shows the detachment of the intimal layer) and its corresponding 3D reconstruction image. Reprinted with permission from Saam et al. (2013) [31].

The X-ray utilized for clinical angiography is insufficient to provide a high-quality image directly, necessitating the administration of a large amount of agent (such as iodine), causing patients discomfort and radiation damage during imaging. Using synchrotron X-ray combination with the K-edge subtraction (KES) method, high-resolution angiographic images can be easily created with a modest amount of contrast agent and a low radiation dose [30]. Usually, this process can be simply described as the following steps. After intravascular injection of low-dose iodine, two images can be recorded in sequence with X-ray beams of different energies: one is above the energy value of the absorption edge K of iodine element, while the other is slightly lower. Due to the absorption difference, the high contrast of vascular images can be obtained by subtracting the two images. Thus, the vessels can be distinguished and quantified. Coronary angiography is one of the few reported applications of synchrotron radiation in human bodies, and it can be used to diagnose diseases such as atherosclerosis [31], arterial embolism [32], and tumor angiogenesis [33]. Figure 3a,b show the schematic drawing and photograph of the imaging set-up at the synchrotron beamline ID19, ESRF, France. Figure 3c,d show a comparison of angiographic images obtained from conventional and synchrotron X-rays. Finer structures (such as the intimal layer of distal external carotid artery) can be resolved by synchrotron X-ray imaging. Meanwhile, it can achieve very high-sensitivity detection with high-resolution images at low agent concentrations and radiation doses. Due to its low invasiveness, synchrotron X-ray coronary angiography has become a helpful technique for fundamental research of cardiovascular disease and its long-term follow-up monitoring after treatment. As a result, clinical trials of coronary angiography were conducted in the 1990s at SSRL (Stanford, CA, USA), Hasylab (Hamburg, Germany), NSLS (Brookhaven, NY, USA), ESRF (Grenoble, France), and KEK (Tsukuba, Japan), where approximately 500 volunteer patients were examined for this technology [34,35]. The results showed that synchrotron radiation-based coronary angiography trials have demonstrated improved spatial resolution (up to 50 μm vs. 200 μm in conventional systems) and reduced radiation exposure (by 30–50%), enhancing diagnostic accuracy and patient safety. The innovative approach to non-invasive and quick examination wins widespread acceptance among volunteer patients and clinicians. Additionally, tumor angiogenesis is a key feature of the progression of malignant tumors and serves as an essential marker for early detection of tumors, the study of tumorigenesis mechanism, and evaluation of curative effects [36]. Synchrotron X-ray phase-contrast imaging offers a high spatial resolution up to the micron level and is also likely to be a valuable tool in the study of early-stage tumor angiogenesis. By using phase-contrast computed tomography, Xuan et al. achieved 3D visualization and morphological quantification of ex vivo liver fibrosis microvessels at different stages at SSRF’s BL13W1 beamline [37]. At present, a large number of anti-tumor angiogenesis drugs (such as axitinib, bevacizumab, cabozantinib, etc.) have been widely applied in the treatment of tumors [38]. Synchrotron X-ray angiography offers a more accurate visualization method for clinical applications such as coronary disease, tumor microvascular identification, and antiangiogenic treatment efficacy evaluation [39]. These advancements underscore the unique capabilities of synchrotron X-ray angiography, as demonstrated by its ability to achieve high-resolution imaging, reduced radiation exposure, and enhanced diagnostic precision, which are well-documented in the literature and clinical trials.

### 3.2. Lung Function Imaging

**Figure 4:**
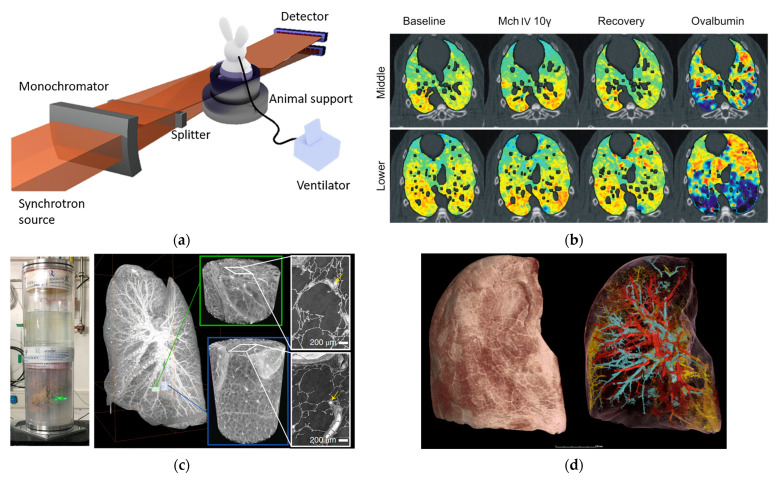
(**a**) Schematic of the set-up of lung synchrotron X-ray imaging [41]. (**b**) Imaging of allergen-induced airway constriction changes in model rabbits [44]. (**c**) Synchrotron X-ray hip-CT imaging of lung tissue ex vivo from a deceased COVID-19 patient. (**d**) High-resolution three-dimensional reconstructed images of lung tissue and major vessels [45]. Reprinted with permission from Bayat et al. (2021) [41]; Bayat et al. (2009) [44] and Walsh et al. (2021) [45].

Lung cancer remains the leading cause of cancer-related deaths in both men and women, accounting for approximately 1.8 million deaths worldwide in 2020 [40]. Early detection and therapy are critical to increasing lung cancer survival rates. However, the heterogeneity in lung structure and function makes it difficult to complete accurate detection by conventional physiological measurement methods. Based on experience with coronary angiography, the synchrotron X-ray KES method can also be applied to lung studies using xenon as a contrast agent [41]. This method enables quantitative visualization of regional lung ventilation, assessment of lung morphology, inflammation, and biomechanics in high-resolution images. The KES technique can be used to image xenon during consecutive breaths and quantify the distribution of ventilation in specific regions. The lung ventilatory heterogeneity affects the matching of regional ventilation and perfusion, resulting in reduced gas exchange efficiency, and can significantly affect the apparent degree of mechanical lung obstruction, which is a pathological feature of many lung diseases [42]. Figure 4a shows a schematic of the set-up of lung imaging in synchrotron radiation beamline station for small animals [43]. Figure 4b shows the lung images of rabbits allergic to ovalbumin after injection of acetylcholine and ovalbumin. The images show that acetylcholine mainly causes the contraction of the central airway in the control group, while more uneven distribution of peripheral ventilation in the lung is visualized in the experimental group [44]. It is caused by contracting the peripheral airway. In addition, phase contrast imaging can be used to improve the resolution of biological soft tissue imaging due to the high coherence of synchrotron X-rays. Phase contrast imaging visualizes structural details in the lungs to a resolution of 1 μm, which is hard to achieve with conventional X-ray absorption contrast imaging. Walsh et al. [45] successfully obtained high-resolution three-dimensional images of isolated lung tissue of COVID-19 patients (Figure 4c,d) using hierarchical phase-contrast tomography (Hip-CT) on beamline BM05 (ESRF), from which the complex pulmonary vasculature could be assessed at micron level. The new technology helps reveal the pathological changes caused by COVID-19 in tiny vessels in lungs. High-resolution imaging can visualize small lesions and provide opportunities for early intervention, improving greatly the recovery rate of lung cancer and the five-year survival rate of patients. At present, synchrotron X-ray imaging has been validated in animal models of different lung diseases and in vitro tissue studies, and feasible imaging schemes have been optimized, but it has not yet entered the clinical stage. The key restrictions are the minimal number of synchrotron radiation sources, the lag of medical imaging beamlines, and the uncertainty of clinical translation radiation safety dosage. Synchrotron X-ray imaging has the capacity to examine the microstructure and functional changes of the lung, and it is predicted to become a valuable imaging tool for early-stage lung cancer detection [46,47,48]. These findings illustrate the potential of synchrotron X-ray imaging in achieving unprecedented visualization of lung microstructures and functional changes, offering a promising avenue for early lung cancer detection and intervention, as highlighted by recent studies and advancements.

### 3.3. Breast Mammography

**Figure 5:**
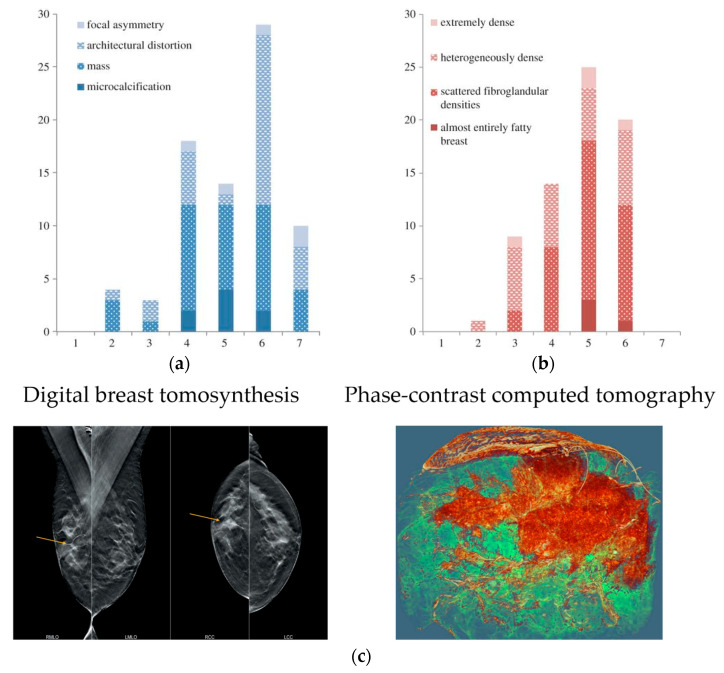
(**a**,**b**) Histogram of relative visibility scores of different types and glandular structures by conventional X-ray mammography and by synchrotron X-ray imaging at the same radiation dose [49]. (**c**) Images of the right breast tissue (ex vivo) obtained from the standard X-ray CT image (**left**) and the synchrotron X-ray PB-CT (**right**). Green to red indicates low to high-density changes in the tissue associated with tumor characteristics [50]. Reprinted with permission from Longo et al. (2014) [49] and Pacilè et al. (2018) [50].

In 2021, there were 9.23 million new cases of cancer in women globally, accounting for 48% of the total new cases in the world. Breast cancer accounted for 2.26 million new cases, much outnumbering other types of cancer [36]. Early detection and treatment of breast cancer have been shown in numerous clinical trials to significantly improve patient survival. The standard screening method for breast cancer is X-ray mammography, but its effectiveness is limited by the overlapping effect of two-dimensional projection images, which can mask tumor features and result in misdiagnosis. Moreover, conventional X-ray mammography has low sensitivity for dense breast tissue and requires compression during examination. Breast cancer imaging was among the initial medical applications of synchrotron X-rays. In 2006–2009, the first clinical studies of phase-contrast mammography were performed at the beamline of the Synchrotron Radiation for Medical Physics (SYRMEP) of Elettra in Italy [49]. This study included 71 patients who had breast abnormalities that remained undiagnosed after routine X-ray and ultrasound examinations at a hospital radiology department. Figure 5a,b present histograms comparing the scores of standard X-ray examination and synchrotron X-ray examination. A score of 7 or higher indicates that the auxiliary diagnostic results obtained through synchrotron X-ray imaging are superior to those of the standard X-ray examination. In general, synchrotron X-ray contrast imaging substantially enhanced the visibility of breast abnormalities and glandular structures while reducing the occurrence of false positives. Three-dimensional X-ray CT imaging necessitates a relatively high radiation dosage, making it crucial to establish a safe radiation dose for clinical purposes. In 2018, Serena et al. developed a propagation-based phase-contrast computational tomography technique (PB-CT) at the imaging and medical beamline (IMBL) of the Australian Synchrotron, with which the imaging of breast cancer tissue ex vivo was realized [50]. Figure 5c shows the comparison of digital breast tomosynthesis images and PB-CT from a mastectomy sample of a 60-year-old patient. Under the same radiation dose of imaging to that of the standard X-ray examination and tomography, the three-dimensional rendering of PB-CT could realize the global analysis of tumors, and better characterize the parameters of tumor shape, boundary, and heterogeneity. The diagnostic accuracy based on PB-CT is much higher than that of conventional X-ray CT at the same radiation dose. The features of these imaging results can be analyzed and extracted, providing a variety of precise parameters for the assessment of clinical tumor characteristics. These parameters are the reliable basis for the accurate diagnosis of multiple lesions in preoperative protocols and even the assessment of changes during chemotherapy. Furthermore, breast cancer images obtained by synchrotron X-ray have the potential to be the gold standard for imaging diagnosis. Clinical X-ray diagnostic instruments can also benefit from medical images with improved resolution and contrast produced by synchrotron X-ray [51,52]. The advancements, including enhanced contrast, improved resolution, and reduced false positives, highlight its potential to revolutionize breast cancer diagnostics and serve as a benchmark for future clinical imaging.

### 3.4. Bone Microtomography

**Figure 6:**
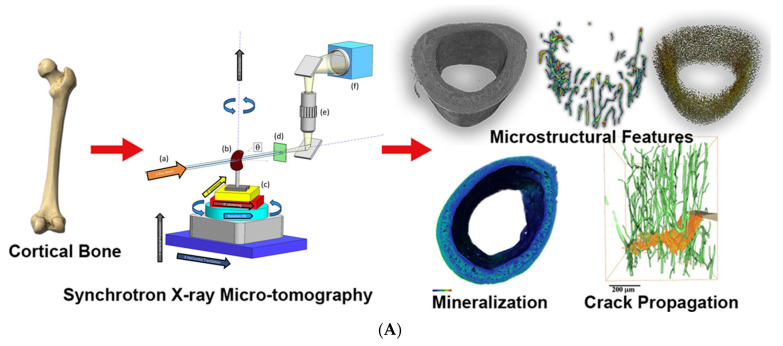

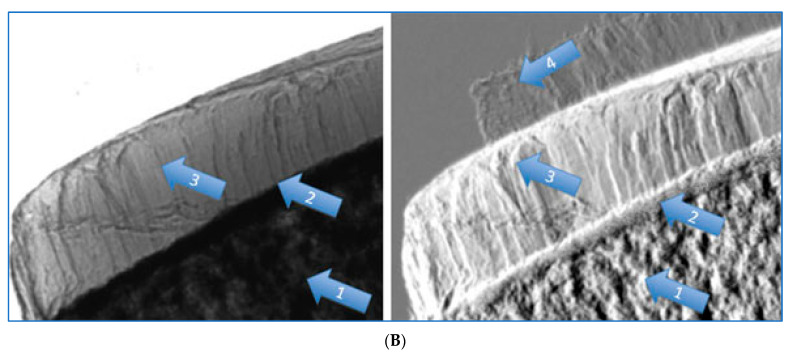
(**A**) High-precision qualitative and quantitative analysis protocol of bone samples with synchrotron X-ray micro-CT [53]. The X-ray beam (a) originating from the synchrotron source passes through the sample (b) mounted on top of precision stage (c), creating a transmission image on the scinitallator (d). The scintillator is crystaline material that converts the X-ray image into visible light which is then imaged directly with a conventional microscope objective (e) and a digital camera (f); (**B**) DEI absorption and refraction images of a human femoral head specimen [56]. Arrows correspond to 1: trabecular bone; 2: subchondral bone; 3: vertical striations; 4: tape/parafilm; Reprinted with permission from Obata et al. (2020) [53] and Muehleman et al. (2004) [56].

Bone possesses a complex hierarchical structure. Gaining a deeper understanding of the correlation between bone structure and mechanical properties holds great potential to aid clinicians in assessing the vulnerability of patients prone to fractures, as well as initiating preventive treatment. Synchrotron X-ray technology lays the foundation for achieving enhanced temporal and spatial resolution in bone imaging. At present, various micro-CT techniques based on synchrotron X-ray have been developed, which can provide information about bone microstructure, ultrastructure, mineralization, and chemical composition. Synchrotron X-ray micro-CT enables the assessment of bone mineralization and microstructure in three-dimensional trabecular or cortical bone on a 5–10 µm scale. On a more microscopic scale of 0.5–1.5 µm, the relevant bone ultrastructure can be further observed [53,54]. In addition, spectroscopic techniques based on synchrotron X-ray are ideal for studying changes in bone composition under physiological or pathological conditions [55]. X-ray phase-contrast imaging can produce higher contrast for soft tissue than attenuation-based conventional X-ray radiology, of which one is diffraction-enhanced imaging (DEI). Figure 6B shows the DEI images of a human femoral head specimen obtained at the SYRMEP beamline of the Elettra light source [56]. The images can directly distinguish vertical stripes of cartilage tissue. In the refraction image (Figure 6B, right), structures of subchondral bone, trabecular bone, and cartilage are simultaneously visible. With relatively low radiation dosages and better image quality than traditional diagnostic techniques, DEI technology can be used to image human cartilage and joints, which is predicted to be applied to the early identification of arthritis and cartilage defects brought on by degenerative diseases [57]. The combination of synchrotron X-ray bone imaging and detection technology can achieve multi-level analysis of bone characteristics and provide a new perspective for bone research. Due to the high radiation dose, the method cannot be applied directly in vivo, but will allow researchers to conduct the study of bone quality ex vivo, including microstructure, fracture mechanisms, and mineralization. It aids physicians in developing novel methods of diagnosis, identifying new treatment objectives, and enhancing the results of treatments. The care and quality of life of patients with skeletal illnesses are subsequently improved [58,59]. These studies demonstrate the potential of synchrotron X-ray imaging in enabling detailed analysis of bone quality and microstructure, providing invaluable insights for skeletal research and paving the way for innovative approaches to diagnosing and treating bone-related diseases.

## 4. Synchrotron X-Ray Therapy

### 4.1. Microbeam Radiotherapy

**Figure 7:**
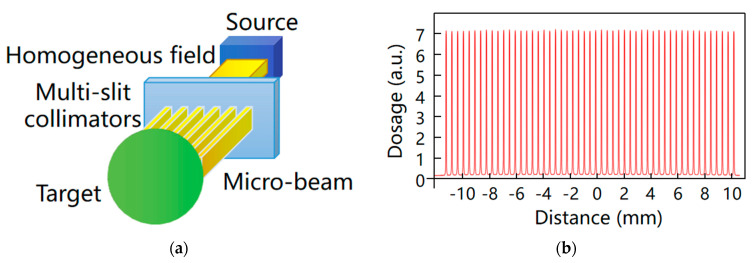
(**a**) Schematic of microbeam irradiation and (**b**) typical microbeam profile [68].

The goal of radiation therapy is to selectively deliver high doses of radiation to local tumors while minimizing the damage to normal tissues. In clinical practice, how to effectively improve the radiation efficiency at tumor regions is an urgent task. Microbeam radiotherapy (MRT) in synchrotron radiation is a new tumor treatment protocol, in which high-intensity synchrotron X-ray beams are generated and the spatial division of beams is realized with multi-slit collimators (the typical width of a single microbeam is tens of microns, and the spacing is hundreds of microns), thereby creating a narrow beam plane with a high radiation dose to ablate the target tumor (Figure 7a). At present, the mechanism of MRT treatment remains to be further elucidated, which may involve the destruction of tumor blood vessels [60], inflammation [61], and activation of anti-tumor immune response [62]. As the tissue positioned in the beam plane between the microbeams can tolerate an increase in radiation dosage, many preclinical types of research have shown that MRT can significantly reduce normal tissue damage while improving the efficiency of tumor radiation therapy [63,64].

In recent years, researchers have verified the feasibility of MRT from preclinical experiments in different animal models. Potez et al. applied MRT for the treatment of melanoma. MRT treatment exhibits a greater tumor vascular destruction impact when compared to traditional radiation therapy and can facilitate the infiltration of immune cells in the tumor site, which results in a better effect of tumor ablation and growth inhibition [62]. Eling et al. treated rats with glioma (9LGS) in conventional wide-beam therapy and MRT in the ERSF-ID17 beamline, and found a nonlinear relationship between the tumor suppression effect and the number of MRT beam trajectories [65]. At the same dose, MRT showed advantages in inhibiting the growth of intracranial tumors and improved the median survival time of tumor-bearing rats. In addition, the blood–brain barrier (BBB) is an important reason for the inefficient delivery of most chemotherapeutic drugs to intracranial tumors [66]. MRT could increase vascular permeability of BBB to enhance drug enrichment in the tumor site. Bouchet et al. used magnetic resonance imaging to compare the BBB changes caused by conventional radiation therapy and MRT [67]. They demonstrated that the increase in tumor vascular permeability caused by MRT was more significant than that caused by conventional therapy. This was shown in earlier and longer-lasting therapeutic responses, as well as increased cell proliferation in the tumor site.

The study of MRT in animal models has become more and more mature. The application of fundamental science to the clinical setting should improve the dosage regimen to ensure safety and effectiveness. For example, at the IMBL in Australia for synchrotron imaging and ID17 beamline in ESRF, scientists constructed customized experimental systems for preclinical MRT studies [68]. Both platforms met the animal research requirements, which ranged from mice to small pigs (<20 kg). To ensure therapy efficacy and safety, MRT development for clinical settings necessitates routine pre-treatment dosimetry assurance. The radiation dose must be maximized in the central area of the planar beam, while the energy deposition is minimized in the area between the beams (Figure 7b). The peak-to-valley dose ratio (PVDR) is one of the key factors. The difference between the peak and valley doses can vary by thousands of Grays over a micrometer distance, and the dose distribution within the region is also affected by the incident X-ray energy and spectrum. There is currently no consensus on the ideal X-ray energy for clinical MRT. However, depending on the application and radiation geometry, the predicted energy range is between 90 and 300 keV [69,70]. Lloyd et al. [71] proposed a method that combines Monte Carlo simulation and a convolution-based method to simulate and calculate the MRT dose, allowing clinicians to understand the possible dose distribution of MRT and decide on the optimal dose for phase I clinical trials. Accurate and personalized treatment is a future development goal of the MRT. Elette et al. [72] combined dosimetry, Monte Carlo simulation, and image guidance to conduct precise MRT in vitro and in vivo, and verified the tumor suppression effect and normal tissue recovery after MRT by using histological staining. Accurate measurement and characterization of the microbeam dose distribution are critical to the efficacy of MRT. Several dosimetry detection techniques have been developed, including Gafchromic films, MOSFET detectors, and silicon detectors [73,74,75]. In addition, conventional X-ray imaging is also impacted by motion artifacts from breathing and heartbeat. The high dose rate of synchrotron radiation beams speeds up imaging significantly, eliminates motion artifacts, and makes X-ray image-guided radiation therapy a possibility. Microbeam diffusion can result in placement mistakes since the tissue moves synchronously with the heart, yet extremely rapid dose delivery assures that the tumor site receives radiation at the micron level. The optimization of dosimetry, imaging guidance, and treatment strategy is required to enable MRT in clinical tumor therapy. More preclinical studies in medical stations are imperative for promoting MRT in the clinic [76,77,78]. The continued advancements in MRT research demonstrate its potential to revolutionize tumor therapy by enabling precise, high-dose tumor ablation with minimal damage to surrounding tissues, paving the way for clinical translation.

### 4.2. Stereotactic Radiotherapy

Synchrotron stereotactic radiotherapy (SSRT) is based on local drug absorption of high heavy-element content in the tumor followed by stereotactic irradiation with low or medium-energy X-rays to enhance radiation dose deposition within the tumor only [79]. Since June 2012, ESRF and the University Hospital of Grenoble (France) carried out the first clinical application study on contrast-enhanced SSRT and evaluated its feasibility and safety. The treatment protocol adopted by ESRF is based on stereotactic radiation, using a high-flux, quasi-parallel X-ray beam (80 keV) to irradiate tumors with high uptake of iodinated agents. The X-ray irradiates the heavy atoms accumulated in the tumor area. Then, the local energy deposition is boosted as a result of the increase in photoelectric cross-section effect, demonstrating a differential irradiation impact between tumor and normal tissue. This allowed for the successful treatment of deeper cancers while maintaining safety [80,81]. SSRT is a technique to deliver high-dose radiotherapy accurately to a small-volume target in a single or a small number of treatment courses, which is mainly used to treat small and well-defined tumors or high-risk areas after dissection, and requires a high-accuracy positioning device. High-dose radiotherapy can be easily achieved by using synchrotron X-ray. Compared with conventional radiotherapy, it realizes the local control of a high radiotherapy radiation dose and has less radiation damage to surrounding normal tissues. In addition, the effect of SSRT can be enhanced by introducing heavy elements such as iodine and platinum. In a mouse brain tumor model, researchers increased the median survival time of tumor-bearing mice by injecting iodine, platinum, or platinized chemotherapy drugs to enhance the therapeutic effect of SSRT [82]. At present, the follow-up time of most SSRT is short, the number of randomized trials is few when comparing SSRT with other treatment options, and its efficacy needs to be further evaluated [83]. SSRT represents a promising approach to precision radiotherapy, offering enhanced tumor targeting and reduced damage to surrounding tissues, with ongoing research paving the way for broader clinical applications in the future.

## 5. Conclusions

Synchrotron radiation facilities play an increasingly essential role in biomedical imaging and radiotherapy. Their ability to generate highly monochromatic and tunable X-rays enables superior imaging quality while minimizing radiation exposure. By mitigating beam hardening artifacts and reducing contrast-agent overdoses, these advantages establish synchrotron-based imaging as a gold standard for early tumor detection and complex diagnostics. Furthermore, the integration of machine learning-driven image processing techniques enhances diagnostic accuracy and efficiency.

In radiotherapy, synchrotron-based approaches, such as microbeam radiation therapy and photon activation therapy, have significantly advanced cancer treatment. These modalities allow for highly targeted tumor ablation while preserving surrounding healthy tissue. The future of oncological treatment lies in the seamless integration of high-resolution imaging with precision radiotherapy, paving the way for more personalized and effective therapeutic strategies.

## 6. Limitations and Future Perspectives

Despite its transformative potential, the clinical implementation of synchrotron radiation remains constrained by several practical challenges. The high operational costs and infrastructural complexity of synchrotron facilities hinder widespread clinical adoption. Additionally, the limited availability of dedicated medical beamlines restricts research throughput and delays clinical translation.

To overcome these barriers, future efforts should prioritize the development of compact, cost-effective synchrotron sources to enhance accessibility. Expanding the number of dedicated medical beamlines will be critical in fostering technological innovation, improving experimental throughput, and accelerating translational research. Interdisciplinary collaboration among physicists, engineers, and biomedical scientists will be essential for optimizing medical end-stations, enhancing imaging resolution, and refining treatment precision. Moreover, advances in computational modeling, next-generation detection technologies, and AI-driven analytics are expected to further accelerate the clinical adoption of synchrotron radiation, ultimately improving diagnostic and therapeutic outcomes.

## Figures and Tables

**Table 1:** Characteristics and medical applications of synchrotron radiation beamlines.

Medical Beamlines	Country	Specification of Device	Featured Applications
European Synchrotron Radiation Facility (ID17)	France	Source: Wiggler Energy range: 25–185 keV Beam size: Min (H × V): 10.0 mm × 51.0 µm Max (H × V): 150.0 mm × 7.0 mm Flux: 2 × 10^14^ ph/s (at 33 keV)	♦ Brain microsurgery [6,7] ♦ Mammography [8] ♦ Cartilage imaging [9] ♦ Functional lung imaging [10] ♦ Microbeam radiotherapy [11,12]
Australian Synchrotron (IMBL)	Australia	Source: Superconducting multipole wiggler Energy range: 25–250 keV Max beam size (H × V): 50 cm × 4 cm bandwidth < 10^−3^ Flux: 3.39 × 10^12^ ph/s (at 22.1 keV)	♦ Lung function [13] ♦ Bone feature measurement [14] ♦ Enhanced Mammography [15] ♦ Microbeam radiotherapy [16,17]
Canadian Light Source (BMIT)	Canada	05 B1-1 beamline Source: Bending magnet Energy range: 12.6–40 keV Beam size (H × V): 200 mm × 4 mm Flux density: 10^9^ ph/s/mm^2^ (mono) 10^12^ ph/s/mm^2^ (pink) 05ID-2 beamline Source: Superconducting wiggler Energy range: 28–140 keV Beam size (H × V): 160 mm × 10 mm Flux density: 5 × 10 ph/s/mm^2^	♦ Visualization of cytoarchitecture [18,19] ♦ Detection of strontium distribution in the osteoporosis model [20,21] ♦ Imaging for bone pore networks [22]
Elettra Sincrotrone Trieste (SYRMEP)	Italy	Source: Bending magnet Energy range: 8–40 keV Beam size (H × V): 160 mm × 5 mm Flux density: 2 × 10^8^ ph/s/mm^2^ (at 20 keV)	♦ Mammography [23,24] ♦ Synchrotron radiotherapy [25]
Super Photon ring-8 GeV (BL20B2, XU)	Japan	Source: Bending magnet Energy range: 5–113 keV Beam size 1 (H × V): 75 mm × 5 mm Beam size 2 (H × V): 300 mm × 20 mm Flux density: 3.6 × 10^8^ ph/s/mm^2^ (at 40 keV)	♦ Bone structure microscopic imaging [26] ♦ Neuron structure analysis [27]
Advanced Photon Source (2-BM-A, B)	America	Source: Bending magnet Energy range: 11–35 keV Beam size (H × V): 25 mm × 4 mm Flux: 1 × 10^12^ ph/s (at 17 keV)	♦ Multi-scale mouse brain imaging [28] ♦ X-ray photodynamic therapy [29]

Note: Data from the official website of the synchrotron radiation facility and published paper.
